# Antibiotic-Resistant and Non-Resistant Bacteria Display Similar Susceptibility to Dielectric Barrier Discharge Plasma

**DOI:** 10.3390/ijms21176326

**Published:** 2020-08-31

**Authors:** Akikazu Sakudo, Tatsuya Misawa

**Affiliations:** 1 School of Veterinary Medicine, Okayama University of Science, Imabari, Ehime 794-8555, Japan; 2 Laboratory of Biometabolic Chemistry, School of Health Sciences, University of the Ryukyus, Nishihara, Okinawa 903-0215, Japan; 3 Department of Electrical and Electronic Engineering, Faculty of Science and Engineering, Saga University, Saga 840-8502, Japan; misawa@cc.saga-u.ac.jp

**Keywords:** antibiotic resistant bacteria, antibiotic resistance gene, disinfection, *E. coli*, inactivation, plasma, sterilization

## Abstract

Here, we examined whether antibiotic-resistant and non-resistant bacteria show a differential susceptibility to plasma treatment. *Escherichia coli* DH5α were transformed with pPRO-EX-HT-CAT, which encodes an ampicillin resistance gene and chloramphenicol acetyltransferase (CAT) gene, and then treated with a dielectric barrier discharge (DBD) plasma torch. Plasma treatment reduced the viable cell count of *E. coli* after transformation/selection and further cultured in ampicillin-containing and ampicillin-free medium. However, there was no significant difference in viable cell count between the transformed and untransformed *E. coli* after 1 min- and 2 min-plasma treatment. Furthermore, the enzyme-linked immunosorbent assay (ELISA) and acetyltransferase activity assay showed that the CAT activity was reduced after plasma treatment in both transformed and selected *E. coli* grown in ampicillin-containing or ampicillin-free medium. Loss of lipopolysaccharide and DNA damage caused by plasma treatment were confirmed by a Limulus test and polymerase chain reaction, respectively. Taken together, these findings suggest the plasma acts to degrade components of the bacteria and is therefore unlikely to display a differential affect against antibiotic-resistant and non-resistant bacteria. Therefore, the plasma method may be useful in eliminating bacteria that are recalcitrant to conventional antibiotic therapy.

## 1. Introduction

In recent years, the emergence of antibiotic-resistant bacteria has become a global problem. One of the main drivers for the emergence of antibiotic-resistant bacteria is the excessive or inappropriate use of antibiotics [1,2].

Clinical isolates of antibiotic-resistant *Escherichia coli* were first isolated in the 1970s [3], soon followed by strains with extended-spectrum β-lactamase (ESBL) genes, which confer resistance to third generation cephalosporins [4], which are the most commonly prescribed classes of antibiotics with bactericidal activity [5]. Multiple resistant bacteria are now a major problem in human and veterinary medicine, especially for nosocomial infections [3,6]. These antibiotic resistance genes are found on plasmids that also carry genes conferring resistance to other non-β-lactam antibiotics such as aminoglycosides and trimethoprim-sulfamethoxazole [4]. *E. coli* harboring these plasmids are only susceptible to carbapenems and colistin.

Antibiotic-resistant bacteria are commonly found in agricultural settings, hospital environments, factory farms, and contaminated foodstuffs [7]. Regardless of the source of antibiotic-resistant bacteria, development of efficient cleaning and disinfection methods are urgently needed to prevent their spread [8].

Several technologies to eliminate antibiotic-resistant bacteria and/or antibiotic resistance genes have been employed including ultraviolet (UV) light [9], solar photo-Fenton process [10], photocatalysis [11], photoelectrocatalysis [12], and nanoparticle treatment [13]. However, some of these technologies have given disappointing levels of disinfection [14,15].

Recently, plasma technologies have been shown to effectively inactivate various bacteria including antibiotic-resistant bacteria [16,17]. Currently, however, it is not known whether the sensitivity against plasma differs between antibiotic-resistant and non-resistant bacteria. Previous studies were conducted in a way that may have masked possible differences arising from sensitivity due to antibiotic resistance. Here, we compared antibiotic-resistant bacteria harboring a plasmid that confers antibiotic resistance with its non-transformed counterpart in terms of susceptibility against plasma generated by a dielectric barrier discharge (DBD) plasma torch. In addition, we analyzed biochemical changes in the bacteria after exposure to the plasma to better understand the mechanism of inactivation.

## 2. Results

**Figure 1:**
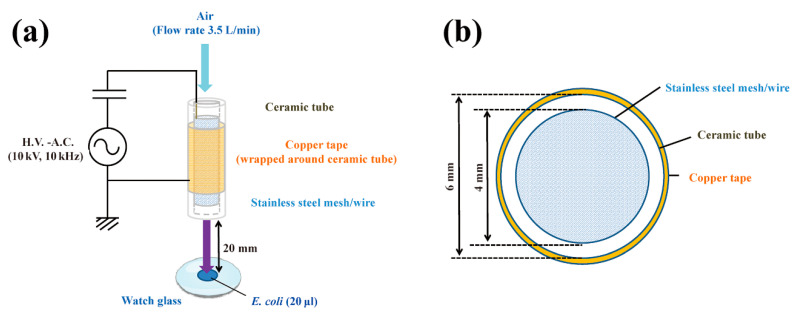
Schematic of a dielectric barrier discharge (DBD) plasma torch. (**a**) The DBD plasma torch comprises a ceramic tube (Al_2_O_3_) (length, 100 mm) containing a stainless-steel mesh (SUS304) and covered with copper tape (thickness, 80 μm) on the outside. The copper tape and stainless steel mesh with stainless steel wire were connected to a power supply (10 kV, 10 kHz); (**b**) Cross-sectional view of the torch, which is made up of a ceramic tube (inner diameter, 4 mm; outer diameter, 6 mm). During gas plasma generation, air flow was maintained at 3.5 L/min using an air pump (Suishin SSPP-2S; Suisaku Co., Sakai, Japan). A suspension (20 µL) of *Escherichia coli* was dropped onto a watch glass. The distance from the top of the plasma torch to the liquid surface on the watch glass was fixed at 20 mm.

**Figure 2:**
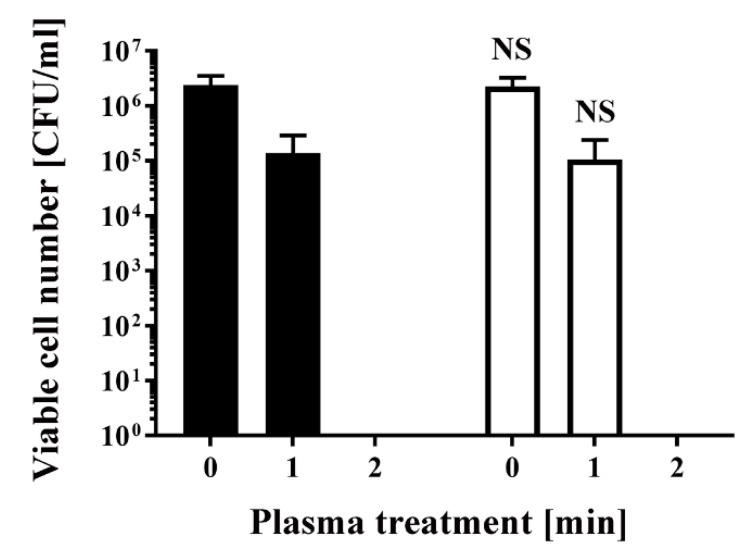
Treatment with a DBD plasma torch reduced the viable cell number of *Escherichia coli* transformed with a plasmid conferring ampicillin resistance, which was selected by spreading on ampicillin-containing agar medium and proliferated in ampicillin-containing liquid medium (■) by a similar amount as plasmid-transformed *E. coli* proliferated in ampicillin-free liquid medium (□). A suspension of *E. coli* transformed with an ampicillin resistance gene-containing plasmid pPRO-EX-HT-CAT, selected in ampicillin-containing agar medium, and proliferated either in ampicillin-containing liquid medium (■) or ampicillin-free liquid medium (□) was exposed to a DBD plasma torch for the indicated time (min). Viable cell count (colony forming units (CFU)/mL) was then determined before and after treatment. NS means no significant difference between ■ and □ at each time when verified by the non-repeated measured ANOVA (analysis of variance) followed by the Bonferroni correction. ANOVA for non-repeated measures was used for comparing the intragroup, while the Bonferroni correction was used as post-hoc analysis.

To obtain antibiotic-resistant bacteria, *Escherichia coli* DH5α was transformed with pPRO-EX-HT-CAT, which contains an ampicillin resistance gene and chloramphenicol acetyltransferase (CAT) gene. Colonies were picked after overnight selection on Luria-Bertani (LB) agar medium containing ampicillin. The selected colonies were subsequently cultured in LB liquid medium with or without ampicillin for 24 h. Aliquots of the resultant *E. coli* were then treated with a dielectric barrier discharge (DBD) plasma torch (Figure 1). As a control, untransformed *E. coli* were also analyzed. The viable cell counts of all *E. coli* samples decreased after plasma treatment. The viable cell number for *E. coli* transformed/selected with pPRO-EX-HT-CAT and cultured in LB liquid medium supplemented with ampicillin was lower than the control (2.40 × 10^6^ ± 1.38 × 10^6^ CFU/mL, 0 min) (Figure 2). Plasma treatment for 1 min reduced viable cell count to 1.38 × 10^5^ ± 0.80 × 10^5^ CFU/mL. However, cultures of the transformed and selected *E. coli* in ampicillin-free LB liquid medium were 2.27 × 10^6^ ± 1.31 × 10^6^ CFU/mL (0 min) and 1.06 × 10^5^ ± 0.61 × 10^5^ CFU/mL (1 min). In summary, no significant difference in viable cell count was observed between the ampicillin-containing culture and ampicillin-free culture. This result suggests that selection pressure with ampicillin does not change the susceptibility of the bacteria to plasma treatment.

**Figure 3:**
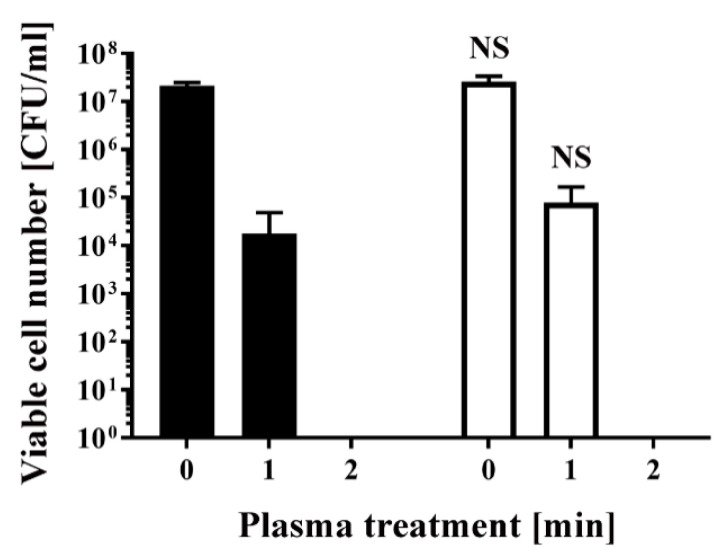
Treatment with a DBD plasma torch reduced the viable cell number of *E. coli* transformed with a plasmid conferring ampicillin resistance, which was selected by spreading on ampicillin-containing agar medium and proliferated in ampicillin-free medium (■), compared with non-transformed *E. coli* proliferated in ampicillin-free medium (□). A suspension of *E. coli* transformed with an ampicillin resistance gene-containing plasmid pPRO-EX-HT-CAT and selected in ampicillin-containing agar medium and proliferated in ampicillin-free liquid medium (■) as well as non-transformed *E. coli* proliferated in ampicillin-free liquid medium (□) was exposed to a DBD plasma torch for the indicated time (min). Viable cell count (colony forming units (CFU)/mL) was then determined before and after treatment. NS means no significant difference between ■ and □ at each time when verified by the non-repeated measured ANOVA followed by the Bonferroni correction.

Similarly, *E. coli* transformed/selected with pPRO-EX-HT-CAT and non-transformed *E. coli* were cultured in ampicillin-free LB liquid medium (Figure 3) and then subjected to plasma treatment. Viable cell number was 2.13 × 10^7^ ± 0.20 × 10^7^ CFU/mL (0 min) and 1.80 × 10^4^ ± 1.80 × 10^4^ CFU/mL (1 min) for the transformed *E. coli*, and 2.59 × 10^7^ ± 0.47 × 10^7^ CFU/mL (0 min) and 7.83 × 10^4^ ± 5.17 × 10^4^ CFU/mL (1 min) for the non-transformed *E. coli,* indicating no significant difference between the two groups. In all cases, the number of viable bacteria was below the detection limit after 2 min treatment (Figure 2 and Figure 3).

**Figure 4:**
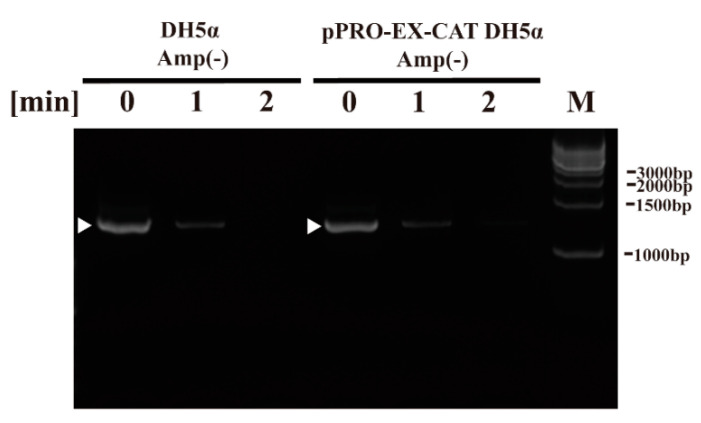
Genomic DNA (16S rDNA) of non-transformed *E. coli* proliferated in ampicillin-free medium (DH5α Amp(−)) and *E. coli* transformed with a plasmid conferring ampicillin resistance, selected in ampicillin-containing agar medium and proliferated in ampicillin-free medium (pPRO-EX-CAT DH5α Amp(−)), was degraded by DBD plasma torch treatment. A suspension of non-transformed *E. coli* proliferated in ampicillin-free medium (DH5α Amp(−)) and *E. coli* transformed with an ampicillin resistance gene-containing pPRO-EX-CAT plasmid, selected in ampicillin-containing agar medium and proliferated in ampicillin-free liquid medium (pPRO-EX-CAT DH5α Amp(−)), was exposed to a DBD plasma torch for the indicated time (min). Viable cell count (colony forming units (CFU)/mL) was then determined before and after treatment. Bands corresponding to amplified *E. coli* 16S rDNA are indicated by arrowheads. Bands corresponding to a DNA size ladder (M) are labelled on the right-hand side of the gel.

Next, we aimed to examine the effect of plasma treatment on components of *E. coli* using the DBD plasma torch. To this end, we performed DNA analysis by polymerase chain reaction (PCR), CAT quantification by ELISA and CAT activity using an acetyltransferase activity assay as well as lipopolysaccharide (LPS) analysis by the Limulus test. PCR amplification using sequence-specific primers for bacterial 16S rDNA followed by agarose gel electrophoresis gave a discrete band in the untreated *E. coli* sample (0 min). DNA sequencing of the amplified band verified that it corresponded to *E. coli* DH5α 16S rDNA (i.e., 99.25 ± 0.35% [5′→3′ direction, *N* = 4] and 99.48 ± 0.34% [3′→5′ direction, *N* = 4] identical to Genbank accession number NZ_CP026085.1) (Figure 4). A band was detected in *E. coli* samples (0 min) in DH5α Amp(−) (non-transformed *E. coli* proliferated in an ampicillin-free LB liquid medium) and pPRO-EX-CAT DH5α Amp(−) (*E. coli* transformed with pPRO-EX-CAT, selected in ampicillin-containing LB agar, and proliferated in ampicillin-free LB liquid medium), although the levels of intact DNA was low at 1 min and undetectable at 2 min for both *E. coli* samples.

**Figure 5:**
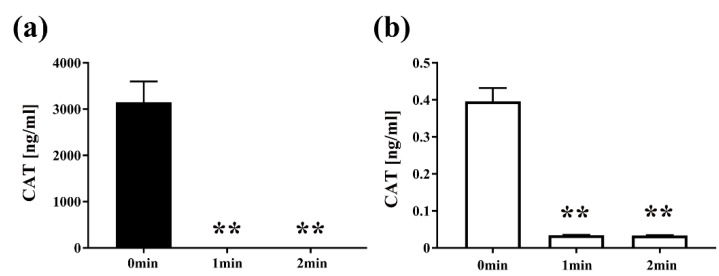
The amount of chloramphenicol acetyltransferase (CAT) in *E. coli* transformed with a plasmid conferring ampicillin resistance, selected on ampicillin-containing agar medium and then proliferated in liquid medium with ampicillin (■) or without ampicillin (□), was reduced by DBD plasma torch treatment. *E. coli* transformed with an ampicillin resistance gene- and CAT gene-containing plasmid pPRO-EX-HT-CAT and selected on ampicillin-containing LB agar were proliferated in ampicillin-containing liquid LB medium (Amp(+)) (■) (**a**) or ampicillin-free liquid LB medium (Amp(−)) (□) (**b**) and then subjected to DBD plasma torch treatment for 0–2 min. The concentration of CAT (ng/mL) was subsequently measured by enzyme-linked immunosorbent assay (ELISA). Differences where *p* < 0.01 (**) versus control (0 min) were significant when verified by the non-repeated measured ANOVA followed by the Bonferroni correction.

Next, to examine the effect of plasma on the quantity of protein, *E. coli* transformed with pPRO-EX-HT-CAT containing CAT gene was treated with the DBD plasma torch and analyzed by CAT ELISA (Figure 5). After exposure to the plasma torch, CAT was reduced in *E. coli* for both transformed and selected *E. coli* cultured in LB medium with or without ampicillin. For *E. coli* transformed with the plasmid and proliferated in the ampicillin-containing medium, CAT concentration before gas plasma treatment was 3147.7302 ± 224.9762 ng/mL (0 min), but was significantly reduced to 0.0325 ± 0.0001 ng/mL and 0.0335 ± 0.0009 ng/mL after plasma treatment for 1 min and 2 min, respectively. In addition, significant reduction of CAT concentration to 0.0344 ± 0.0004 ng/mL at 1 min and 0.0339 ± 0.0004 ng/mL at 2 min was also observed in transformed *E. coli* cultured in ampicillin-free medium compared to the control of 0.3961 ± 0.0182 ng/mL at 0 min. In summary, a significant decrease in the amount of CAT for the transformed *E. coli* was observed at each time point compared to 0 min.

**Figure 6:**
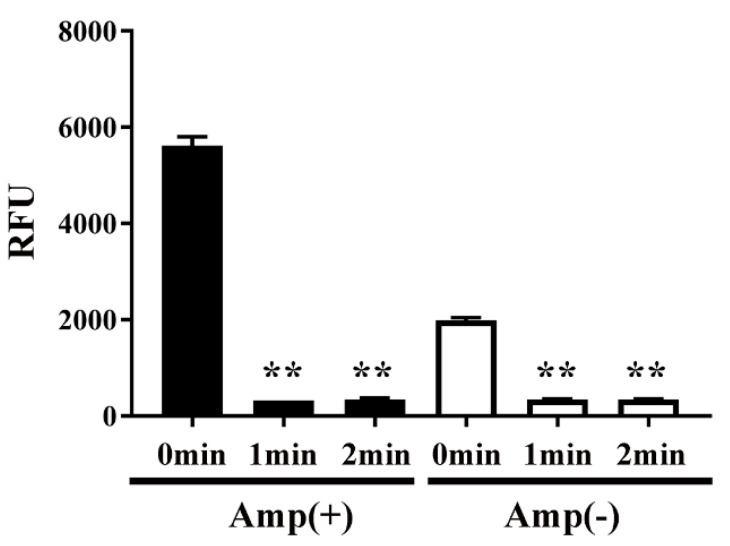
CAT activity in *E. coli* transformed with an ampicillin resistance gene- and CAT gene-containing plasmid, selected on ampicillin-containing agar medium, and proliferated in liquid medium with ampicillin (■) or without ampicillin (□) was decreased by DBD plasma torch treatment. *E. coli* transformed with an ampicillin resistance gene- and CAT gene-containing plasmid pPRO-EX-HT-CAT and selected on ampicillin-containing medium were proliferated in ampicillin-containing liquid medium (Amp(+)) (■) or ampicillin-free liquid medium (Amp(−)) (□) and then subjected to DBD plasma torch treatment for 0–2 min. The activity of CAT in the bacteria was subsequently measured using an acetyltransferase activity assay kit (Enzo Life Sciences, Inc.) with an excitation wavelength of 400 nm and emission wavelength of 490 nm. Differences where *p* < 0.01 (**) versus control (0 min) were significant when verified by the non-repeated measured ANOVA followed by the Bonferroni correction.

Next, to examine the effect of plasma on protein function, the activity of CAT in transformed *E. coli* was measured by an acetyltransferase activity assay (Figure 6). We speculated that plasma might interfere with the activity of CAT. Specifically, the activity of CAT in *E. coli* transformed with pPR-EX-HT-CAT, selected in ampicillin-containing LB agar, and then proliferated in ampicillin-containing LB liquid medium was 5617.49 ± 92.17 RFU (0 min), but decreased to 322.22 ± 1.07 RFU and 345.11 ± 10.88 RFU after DBD plasma torch treatment for 1 min and 2 min, respectively. In addition, 1991.12 ± 27.76 RFU (0 min) and 343.10 ± 7.47 RFU (1 min) and 343.27 ± 5.74 RFU (2 min) were also observed in cultured *E. coli* after transformation/selection of pPRO-EX-HT-CAT in the ampicillin-free LB liquid medium. Thus, a significant decrease in CAT activity of the transformed *E. coli* was observed at each time compared to 0 min.

**Figure 7:**
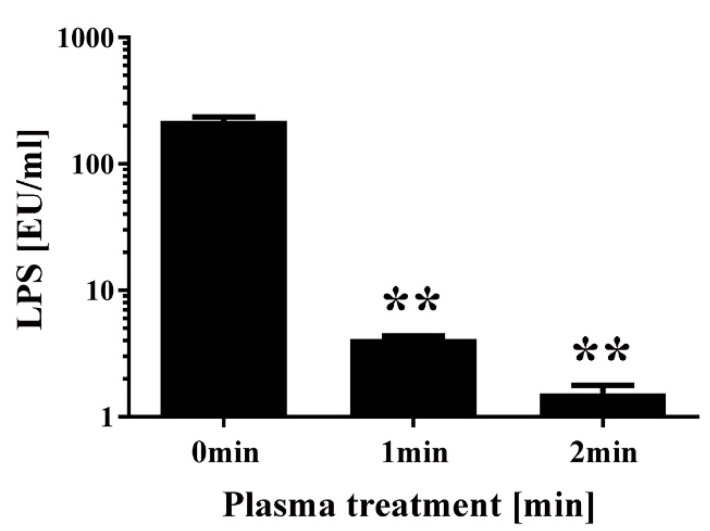
Changes to the level of lipopolysaccharides (LPS) in *E. coli* following DBD plasma torch treatment. The concentration of LPS measured by the Limulus test (Limulus-color KY test, Wako Pure Chemical Industries Ltd., Osaka, Japan) is expressed as endotoxin units (EU) per mL. LPS level was reduced after plasma treatment for 0–2 min. Differences where *p* < 0.01 (**) versus control (0 min) were considered significant when verified by the non-repeated measured ANOVA followed by the Bonferroni correction.

Finally, a chromogenic Limulus test was performed to measure the amount of intact LPS in non-transformed *E. coli* following DBD plasma torch treatment (Figure 7). The results showed that intact LPS in *E. coli* after plasma treatment significantly decreased from 218.94 ± 16.38 EU/mL at 0 min to 4.14 ± 0.22 EU/mL at 1 min and 1.53 ± 0.24 EU/mL at 2 min. These findings indicate that *E. coli* LPS lipid A, which is located on the outer surface of the bacteria, is degraded after plasma treatment. In conclusion, plasma treatment may degrade the integrity of the cell wall of *E. coli*.

## 3. Discussion

The growing risk of disease from antibiotic-resistant bacteria is now a global public health concern [18]. The spread of antibiotic resistance is caused, in part, from the extensive and indiscriminate use of antibiotics, which leads to the emergence of antibiotic-resistant bacteria. To restrict the increase of antibiotic-resistant bacteria, efficient inactivation methods that do not rely on antibiotics are required.

Previous studies have shown that exposure to plasma, which is a state of matter predominantly composed of ions and electrons, can inactivate a variety of antibiotic-resistant bacteria. For example, glow discharge plasma has been shown to inactivate antibiotic resistant *E. coli* [16]. Surface micro-discharge (SMD) plasma treatment was demonstrated to inactivate antibiotic-resistant bacteria including *Yersinia enterocolitica*, *Staphylococcus aureus*, *Klebsiella*, *E. coli*, *Acinetobacter*, and *Enterococcus faecium* [17]. Moreover, a floating-electrode DBD plasma device has successfully inactivated methicillin-resistant *S. aureus* (MRSA) [19]. In addition, suspensions and biofilms of MRSA were inactivated by RF (radiofrequency) plasma [20] and floating-electrode DBD [21]. Therefore, plasma treatment provides a robust and efficient way of eliminating antibiotic-resistant bacteria. However, to date, there are no reports showing whether antibiotic-resistant and non-resistant bacteria display the same or different level of sensitivity to plasma treatment.

In this study, we have clarified that antibiotic-resistant and non-resistant bacteria show no significant difference in susceptibility to treatment with plasma. Furthermore, this study suggests that the emergence of plasma-resistant bacteria following plasma treatment is low.

Our analysis showed a reduction in the levels of CAT, DNA, and LPS in *E. coli* after plasma treatment. Previous studies have analyzed gas plasma [22,23,24,25], which led to oxidation/modification of DNA and protein as well as their degradation. LPS, also termed as endotoxin, is a component of the outer membrane of Gram-negative bacteria and is known to be highly resistant to both physical and chemical treatment [26]. However, a previous study has shown that the level of endotoxin can be reduced by exposure of bacteria to nitrogen gas plasma [27]. Here, we found that LPS is degraded by DBD plasma torch treatment. These findings are consistent with a previous study, in which the authors proposed that cell surface components are important in the mechanism of inactivation because the thickness of the polysaccharide membrane influences sensitivity to plasma [17]. Furthermore, as previously determined [28], the temperature of the liquid surface was 38.40 ± 0.12 °C at the 2 min treatment time using the same DBD plasma torch under identical conditions. *E. coli* can survive at around 38 °C [29]. Thus, heating during operation of the plasma torch cannot have been the cause of *E. coli* inactivation.

To clarify the likely inactivation mechanism, analysis of plasma components by electron spin resonance (ESR) was also performed (Supplementary Figure S1). These experiments showed that OH radicals (OH·) and H radicals (H·) were present in the plasma. Thus, reactive chemical species such as OH and H radicals may act to eliminate bacteria by oxidizing biomolecules. Indeed, these findings are consistent with a previous study that showed that the OH radical is the most important factor for bacterial inactivation during plasma treatment [16,30]. Thus, oxidation and degradation of biomolecules may be caused by reactive chemical species such as reactive nitrogen species (RNS) and reactive oxygen species (ROS) generated by the plasma. Nevertheless, it is possible that the reactive chemical species may act on other biomolecules. In addition, as antioxidants have antimicrobial activity against antibiotic-resistant bacteria [31], modulation of the oxidative conditions might change the antibacterial activity. Thus, further studies on the potential relationship between oxidative modulation by plasma and its antibacterial activity would be necessary to perform.

Regardless of the underlying mechanism of inactivation, the DBD plasma torch was found to be highly effective at eliminating antibiotic-resistant bacteria. Presumably, this was because changes to cell components induced by the plasma treatment such as modifications to the proteins and DNA were independent of antibiotic resistance. Further studies are also needed to clarify the inactivation mechanism of the DBD plasma torch including detailed analysis of bactericidal factors generated by the plasma that induce damage to DNA and proteins as well as cell surface degradation including LPS, which is a component of the outer membrane of Gram-negative bacteria. Antibiotic resistant bacteria employ multiple mechanisms to confer resistance including antibiotic inactivation, target-site modification, and reduction of cytoplasmic antibiotic concentration [32]. The specific type of antibiotic resistance mechanism might be related to plasma susceptibility. Therefore, further studies on the relationship between the various mechanisms of antibiotic resistance and plasma susceptibility are required.

Finally, the development of process compatible technology design is required to convert the DBD plasma device into a commercial and practical technology for disinfection in agricultural settings. To approach this issue, we have recently designed a new type of DBD plasma device (a roller conveyer plasma device) that is well suited to the disinfection of agricultural products such as vegetables and fruits during the sorting process on rollers [33]. To achieve broad applicability for the DBD plasma device across a range of agricultural food products, further development and improvement such as scale-up and better cost-performance will be needed. Thus, further optimization of plasma generation to maximize disinfection efficiency will be required before the device can be widely applied in a commercial setting.

## 4. Materials and Methods

### 4.1. DBD Plasma Torch

The DBD plasma torch used in the present study was the same as described in a previous report [34]. Briefly, a ceramic tube (Al_2_O_3_) (inner diameter, 4 mm; outer diameter, 6 mm; length, 100 mm) was wound around the tube by a copper tape (thickness, 80 µm; length, 60 mm) that was used as an earth electrode (Figure 1). A stainless steel mesh (SUS304) with stainless steel wires was placed inside the tube to act as a high voltage electrode. Next, a low frequency and high-voltage power supply (10 kV peak-to-peak, 10 kHz) was connected to the two electrodes. During plasma generation, an air pump (Suishin SSPP-2S; Suisaku Co., Sakai, Japan) was used at an air flow rate of 3.5 L/min. The distance from the torch top to the liquid surface was set at 20 mm. Aliquots (20 μL) of *E. coli* suspension were dropped on a watch glass and subjected to plasma treatment.

### 4.2. Plasma Treatment of Transformed Bacteria and Colony Counting

*E. coli* (DH5α Competent Cells, Takara Bio, Shiga, Japan) was used for the plasma treatment. Transformation of pPRO-EX-HT-CAT (Thermo Fisher Scientific, Waltham, MA, USA) with a plasmid carrying an ampicillin-resistance gene and chloramphenicol acetyltransferase (CAT) gene was performed according to a conventional methodology [35]. *E. coli* obtained after selection on LB (Luria-Bertani) agar medium supplemented with ampicillin (50 mg/L) were picked and then cultured in LB liquid medium with or without ampicillin (50 mg/L) at 37 °C for 24 h. The resultant *E. coli* were then subjected to plasma treatment. As a control, untransformed *E. coli* were also exposed to plasma. Viable cell count after plasma treatment was performed by spreading onto LB agar medium with or without ampicillin (50 mg/L). After overnight culture at 37 °C, colony counting was performed to determine the colony forming units per mL (CFU/mL).

### 4.3. Enzyme-Linked Immunosorbent Assay (ELISA)

CAT was quantified by ELISA using a CAT ELISA Kit (cat no. #11 363 727 001; Roche, Rotkreuz, Switzerland) in the accordance with the manufacturer’s instructions.

### 4.4. Acetyltransferase Activity Assay

CAT activity was measured using an acetyltransferase activity assay kit (Enzo Life Sciences Inc., Farmingdale, NY, USA) as the index of RFU (relative fluorescence units) with an excitation wavelength of 400 nm and an emission wavelength of 490 nm.

### 4.5. PCR

The level of intact *E. coli* genomic DNA was assessed using a Bacterial 16S rDNA PCR Kit (Takara Bio Inc., Shiga, Japan) according to the manufacturer’s instructions. Bacterial DNA samples were subjected to 30 cycles of PCR amplification conditions (94 °C for 0.5 min, 55 °C for 0.5 min, 72 °C for 1 min) using a PC320 thermal cycler (Astec Co. Ltd., Fukuoka, Japan). Amplified DNA was analyzed by agarose gel electrophoresis (1% gel) and the DNA was visualized using a WSE-5200 Printgraph 2M device (ATTO Corporation, Tokyo, Japan). The amplified products were extracted using a QIAquick Gel Extraction Kit (QIAGEN, Hilden, Germany) and directly subjected to DNA sequencing using primers F1 and R1 (*bacterial*) of the Bacterial 16S rDNA PCR Kit with an ABI 373 OXL Genetic Analyzer (Applied Biosystems, Foster City, CA, USA).

### 4.6. Measurement of LPS

DBD plasma-treated or untreated bacterial suspensions were resuspended in distilled water (Otsuka Pharmaceutical Co. Ltd., Tokyo, Japan) and subjected to the chromogenic Limulus test (Limulus-color KY test; Wako Pure Chemical Industries Ltd., Osaka, Japan) for quantifying LPS. Absorbance at 415 nm by reference to that at 655 nm was compared with a standard curve obtained using a LPS solution (Wako Pure Chemical Industries Ltd., Osaka, Japan). LPS concentrations (Endotoxin Units (EU)/mL) were then estimated from the index of absorbance.

### 4.7. Statistical Analysis

GraphPad Prism 7.02 software (GraphPad Prism Software Inc., La Jolla, CA, USA) was used for the statistical analysis. Mean ± standard deviation of experiments carried out at least in triplicate are shown. Non-repeated analysis of variance (ANOVA) followed by Bonferroni’s multiple comparison test was applied to the statistical analysis of significant difference.

## 5. Conclusions

The present study established that there was no difference in susceptibility between antibiotic-resistant and non-resistant bacteria to plasma treatment using an DBD plasma torch. In addition, ESR has shown that the plasma torch generates reactive chemical species including OH and H radicals, which may contribute to the inactivation of DNA, proteins, and LPS. However, OH and H radicals may react with various other bacterial components to generate oxidation products. Therefore, the bactericidal mechanism needs to be further clarified by the detailed analysis of changes to the components of the bacteria induced by radicals generated by the plasma.

Nonetheless, the mechanism of inactivation is unlikely to differ between antibiotic-resistant and non-resistant bacteria. Therefore, the plasma method may be especially useful for eliminating antibiotic-resistant bacteria from the environment. Data on the likely inactivation mechanism may contribute to improving the efficiency of the plasma system by fine-tuning plasma generation to maximize bactericidal action. Indeed, future optimization of the plasma system based on knowledge gained concerning the inactivation mechanism(s) may facilitate increased inactivation efficiency.

## Supplementary Materials

The following are available online at https://www.mdpi.com/1422-0067/21/17/6326/s1.

## Abbreviations

ANOVA	Analysis of variance
CAT	Chloramphenicol acetyltransferase
CFU	Colony forming units
DBD	Dielectric barrier discharge
ELISA	Enzyme-linked immunosorbent assay
ESBL	Extended-Spectrum β-lactamase
ESR	Electron spin resonance
EU	Endotoxin Units
H·	H radical
LB	Luria-Bertani
LPS	Lipopolysaccharides
MRSA	Methicillin-resistant *S. aureus*
OH·	OH radical
PCR	Polymerase chain reaction
RFU	Relative fluorescence units
SMD	Surface micro-discharge
UV	Ultraviolet
