# Supplementary material for: Antibiotic-Resistant and Non-Resistant Bacteria Display Similar Susceptibility to Dielectric Barrier Discharge Plasma
Source: Int J Mol Sci. 2020 Aug 31;21(17):6326. doi: 10.3390/ijms21176326 (PMC7504529; doi:10.3390/ijms21176326)
Supplement: Supplementary file 1 [file ijms-21-06326-s001.pdf]

Supplementary Materials

Antibiotic-Resistant and Non-Resistant Bacteria Display Similar Susceptibility to Dielectric Barrier Discharge Plasma

Electron spin resonance (ESR) spectroscopy detected radicals generated during DBD plasma torch operation.

Materials and Methods

ESR

OH radicals (OH·) and H radicals (H·) generated in water by plasma irradiation were detected using an electron spin resonance meter (TE-100, JEOL Ltd., Tokyo, Japan). DMPO (5,5-dimethyl-1-pyrroline *N*-oxide) purchased from Kyoto-Spinlabo CO., Ltd. (Kyoto, Japan) was used as a radical trapping agent. The concentration of DMPO was 100 mM in distilled water (DMPO solution). After bubbling with nitrogen gas for 5 min (flow rate: 1.0 L/min) to remove dissolved oxygen from the DMPO water, 1.0 mL of DMPO solution in a 2.0 mL-microtube was subjected to 5 min plasma irradiation using a DBD plasma torch. The distance between the DBD discharge plasma and the surface of the DMPO water was 10 mm. An aliquot of the solution was then collected using a hematocrit tube and placed in a quartz tube for ESR measurements. All ESR measurements were carried out at a microwave frequency of 9.4280 GHz, with a 60 s sweep time and an acquisition frequency of 1 time. Measurement conditions for the ESR device included a magnetic field of 335.6 ± 5.0 mT, modulation width of 0.10 mT, modulation frequency for the magnetic field of 100 kHz, time constant for detection of 30 ms and a gain for the detector of 125–500. Estimations of *g* values were performed using the signal of Mn^{2+} in MgO matrix as a secondary standard.

Results

ESR was used to investigate the reaction process of gas plasma in a DBD plasma torch. The radical scavenger DMPO was used to detect free radicals by ESR. The main component of the ESR spectrum was a strong signal of four lines, giving a signal strength ratio of 1:2:2:1, together with several weaker signals. The strong signal is derived from a DMPO/OH radical in which an OH radical reacts at the 2-position of DMPO, and the weak signal can be attributed to a DMPO/H radical in which a hydrogen atom reacts at the 2-position of DMPO. These results suggest that OH radicals and H radicals were generated by plasma irradiation.

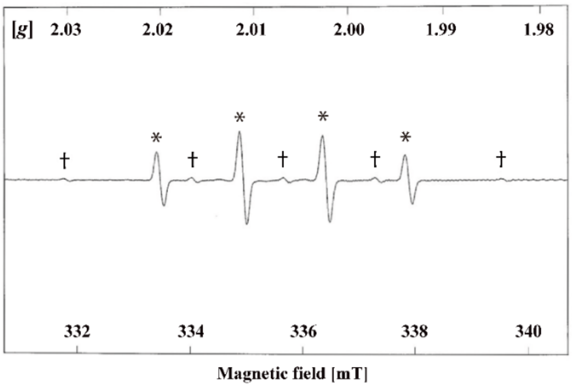

Figure S1. ESR detected radicals generated during operation of a DBD plasma torch. ESR spectra of spin adducts in aqueous solution irradiated using a DBD plasma torch with nitrogen at a flow rate of 1.0 L/min for 10 min after bubbling with nitrogen gas. ESR spectrum of DMPO shows a 1:2:2:1 quartet of DMPO-OH (*) and several other smaller peaks due to DMPO-H (†).
